# Immunotherapy for Alzheimer’s disease: past, present and future

**DOI:** 10.3389/fnagi.2014.00114

**Published:** 2014-06-10

**Authors:** Brian Spencer, Eliezer Masliah

**Affiliations:** ^1^Department of Neurosciences, University of CaliforniaSan Diego, La Jolla, CA, USA; ^2^Department of Pathology, University of CaliforniaSan Diego, La Jolla, CA, USA

**Keywords:** Alzheimer’s disease, immunotherapy, blood-brain barrier, Aβ, immunization

## Abstract

Alzheimer’s disease (AD) is an incurable, progressive, neurodegenerative disorder affecting over 5 million people in the US alone. This neurological disorder is characterized by widespread neurodegeneration throughout the association cortex and limbic system caused by deposition of Aβ resulting in the formation of plaques and tau resulting in the formation of neurofibrillary tangles. Active immunization for Aβ showed promise in animal models of AD; however, the models were unable to predict the off-target immune effects in human patients. A few patients in the initial trial suffered cerebral meningoencephalitis. Recently, passive immunization has shown promise in the lab with less chance of off-target immune effects. Several trials have attempted using passive immunization for Aβ, but again, positive end points have been elusive. The next generation of immunotherapy for AD may involve the marriage of anti-Aβ antibodies with technology aimed at improving transport across the blood-brain barrier (BBB). Receptor mediated transport of antibodies may increase CNS exposure and improve the therapeutic index in the clinic.

## Introduction

Dementia and its associated pathologies are a significant health threat affecting society today. Alzheimer’s disease (AD) is the most common form of dementia and involves the accumulation of intra- and extra- neuronal Aβ as well as intra-neuronal Tau. There are currently more than 5 million AD patients in the United States and over 35 million patients worldwide. This number is expected to double every 20 years due to the increased aging population. AD is the 6th leading cause of death in this country and the only cause of death among the top 10 in the United States that cannot be prevented, cured or even slowed. Based on mortality data from 2000–2008, death rates have declined for most major diseases while deaths from AD have risen 66% during the same period (Alzheimer’s association, 2013).

AD is caused by the progressive accumulation of Aβ into oligomers and plaques leading to synaptic loss, neuronal dysfunction and death. In addition, intra-neuronal Tau aggregates form characteristic neurofibrillary tangles that contribute to neuronal death. Increased immune response by astrogliosis and microgliosis lead to pro-inflammatory cytokine release that contributes to neuronal cell loss (Terry et al., 1994).

**Figure 1:**
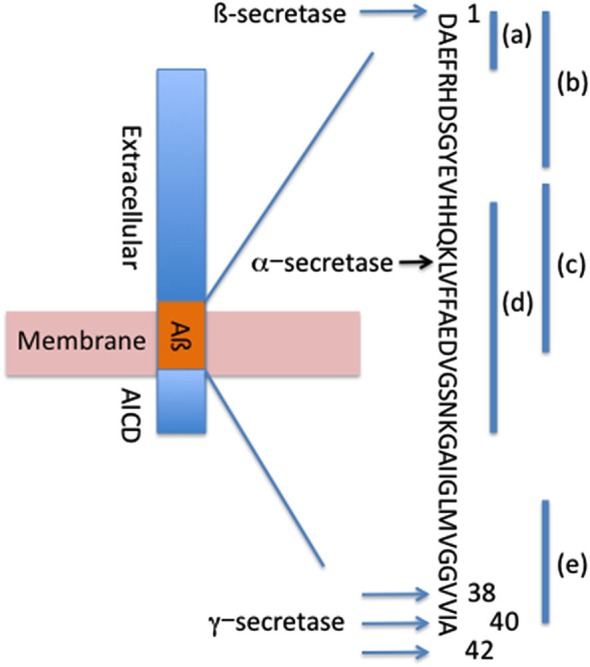
**Systematic processing of APP forms Aβ_42_, Aβ_40_ and Aβ_38_**. Locations of β-secretase, α-secretase and γ-secretase cleavage sites are depicted. Binding sites for passive antibodies are identified as (a)—Bapineuzumab, (b)—Gantenerumab, (c)–Crenezumab, (d)—Solanezumab, (e)—Ponezumab.

Aβ is formed by the systematic processing of the APP transmembrane protein by proteolytic cleavage on the extracellular side of the membrane by β-secretase at the N-terminus of Aβ and by the γ-secretase complex at the C-terminus of Aβ to generate Aβ_38_, Aβ_40_ and Aβ_42_ (Figure 1; Sisodia and St George-Hyslop, 2002). Alternatively, under physiological conditions a significant fraction of APP is cleaved at the middle of Aβ (aa 16) by proteases with α-secretase activity (Anderson et al., 1992; Allinson et al., 2003), such as members of a disintegrin and metalloproteinase (ADAM) family of enzymes (Kojro and Fahrenholz, 2005), and under α-secretase cleavage no Aβ is generated. This cleavage event results in the production of a neurotrophic secreted (s)APP, which acts as a growth factor for many types of cells and promotes neurite outgrowth in post-mitotic neurons (Gralle and Ferreira, 2007).

The formation of Aβ_42_ fragments over Aβ_38_ or Aβ_40_ appears to occur as a function of genetic and/or environmental factors. The Aβ_42_ fragment is considered more toxic due to its ability to readily assemble into oligomers and higher order fibrils which themselves present toxic products to the neuron. Aβ monomers and oligomers can also bind to cell surface receptors promoting signaling pathways and inducing neuronal degeneration (Patel and Jhamandas, 2012).

Aβ_42_ small order oligomers may also propagate from neurons to spread the disease (Harris et al., 2010; Nath et al., 2012). This may explain the progression of the disease and pathological progression of Aβ accumulation and plaque deposition beginning in the entorhinal cortex proceeding to the hippocampus and to cortical areas (Braak and Braak, 1991, 1996). This results in progressive memory and cognitive deficits observed in the patients (Blennow et al., 2006).

To date therapeutic interventions for AD have been primarily limited to treating symptoms without the ability to target the underlying causes of Aβ accumulation. These treatments include acetylcholinesterase inhibitors (donepezil, rivastigmine and galantamine) and N-methylD-aspartic acid (NMDA) glutamate receptor antagonists (memantine) (Tayeb et al., 2012). β-secretase inhibitors have been tested clinically in an attempt to reduce the production of Aβ. These small molecule and peptidomimetic inhibitors have succeeded in reducing plasma and CSF levels of Aβ; however; off-target toxicity and secondary neurodegeneration have been observed (Vassar et al., 2014). Immunotherapy is one method that has been advanced recently for its ability to reduce the accumulation of Aβ and potentially treat the underlying cause of AD.

## Active immunization

Vaccination has been an instrument in the tool chest of the clinician since the late 18th century when Edward Jenner first used the related cowpox virus to immunize patients against small pox. The basic concept of active immunization is to prime the immune system to recognize an antigen as a foreign protein in order to mount a response against it. The most commonly recognized active immunization strategies are used against bacterial (e.g., pertussis, typhoid, meningitis), viral (e.g., influenza, hepatitis, chicken pox) and toxin (e.g., diphtheria, tetanus) antigens.

It has only been recently that investigators have attempted to utilize the human immune system to rid the body of potentially harmful or toxic proteins that are endogenously produced. In this instance, the immune system has initially been taught to ignore the proteins as “self” antigens. Antibody producing cells (B-cells) enter a maturation process that includes removing all cells that could possibly generate antibodies against proteins the body already makes in a process called B-cell tolerance. This ensures that the immune system will not accidentally generate antibodies or mount an immune response against itself. Thus the clinical intervention of inducing an immune response against Aβ (an endogenous protein) would require breaking the self-tolerance.

In addition, it was not entirely clear that it would be possible or even effective to elicit and immune response against a neuro-protein such as Aβ. Two important considerations slowed the entry into the field of vaccination of a neuronal protein: (1) Antibody transport from the blood to the brain was known to be limited; and (2) The brain has long been considered an immune-protected organ in the body. The barrier between the blood and the brain is called the blood-brain barrier (BBB) and prevents the passage of most proteins and small molecules from entering the brain providing a protected environment for the neurons. Only proteins with specific receptors on the BBB are known to actively transport to the neuronal side, and antibody receptors are not known to be expressed on the BBB so significant transport of antibodies could not be assumed to be a given proposition (Hussain et al., 1999). Second, the brain had long been considered an “immune-protected” organ precisely for the fact that few antibodies and immune-modulatory cells transit across the BBB. It was not known that an antibody response could be mounted to a neuronal protein or if one could, what effect it would have.

With these facts in mind, it was a surprise in 1999 when Schenk et al. showed that immunization of the PDGF driven APP (PDAPP) mouse model of AD with Aβ_42_ could prevent the onset of plaque formation if performed in young mice. Immunization of older mice effectively slowed the progression of plaque formation (Schenk et al., 1999). These results were replicated in numerous animal models suggesting that they could be translated to the clinic (Das et al., 2001; Wilcock et al., 2001).

Translation to the clinic was expected to bring a breakthrough in treatment of AD as the first therapeutic to directly target the generation and accumulation of Aβ protein. Clinical trial AN1792 used the full length Aβ_42_ as an active vaccine with an adjuvant to prime the immune system. 372 patients were enrolled; however the trial was suspended at phase IIa when four patients came down with meningoencephalitis. Later analysis showed that of the patients that showed an antibody response to the Aβ antigen, several (seven patients analyzed) showed significant clearing of hippocampal Aβ plaque and reduced plaque density as well as reduced phosphorylated tau compared to non-treated AD patients (Masliah et al., 2005; Serrano-Pozo et al., 2010). Follow-up studies revealed reduced CSF Tau and improved cognitive scores (Boche et al., 2010). Further analysis of the AN1792 trial revealed a T-cell response to the Aβ epitope found at amino acids 25–35 so future immunization strategies have attempted to avoid this epitope (Frenkel et al., 2001).

More recent active immunization strategies have focused on the B-cell epitopes of the Aβ protein located at the N-terminus of the protein while avoiding the T-cell epitopes located at the C-terminus of the protein. These include Affitope; which is composed of a synthetic six amino acid sequence mimic of the N-terminus of the Aβ peptide comprising the B-cell epitope without the C-terminus T-cell epitope (Schneeberger et al., 2009). Affitope is currently in phase II trials.

CAD-106 is comprised of multiple copies of amino acids Aβ1-6 expressed from the virus Qβ. Using the bacteriophage Qβ provides a scaffold for presentation of multiple copies of the Aβ1-6 epitope to a single cell to ensure maximum activation of immune cell response, a necessity when attempting to overcome self-tolerance. Vaccination in mouse models of AD showed reduced CNS Aβ load (Wiessner et al., 2011). Clinical trials show good safety data dose tolerability with antibody production (Lemere and Masliah, 2010; Winblad et al., 2012).

Vanutide cridificar is composed of multiple copies of Aβ1-7 fused to an inactivated diphtheria toxin. Clinical trials show good safety and tolerability with antibody titer production, and trials are currently in phase II (NIH, 2014a).

## Passive immunization

While active immunization followed the well worn path of vaccination that has proven successful in the clinic at preventing and treating diseases that presented as foreign antigens such as bacteria, viruses and toxins; passive immunization has shown more success in treating diseases that present with “self antigens”. In this instance, rather than prime the human immune system to generate and sustain an immune response against a novel antigen, the premise is to identify an epitope in the laboratory, generate antibodies *ex vivo* and then directly inject these antibodies into the patient. The advantages to this approach are directing the epitope to which the antibodies will be targeted, the isotype of antibody generation, the antibody dose delivery and interval. One disadvantage to passive immunization is the possible requirement for continuous dosing of the antibody.

This approach has been successful in the clinic for a number of diseases including autoimmune disorders (Humira, Actemra), cancer (Herceptin, Rituxan) and transplant rejection (Zenapax, Simulect) (Waldmann, 2003). Thus the idea of transferring the knowledge gained from the use of monoclonal antibody therapies for human therapies to neurological disorders such as AD was a natural extension.

Based on the results obtained in the active immunization work, antibodies were developed against the N-terminus of the Aβ protein for direct injection. Treatment of APPtg mouse models of AD with the antibodies showed significant reductions in CNS Aβ and reversed memory deficits in object recognition and Morris water maze (Dodart et al., 2002; Kotilinek et al., 2002; Bard et al., 2003; Buttini et al., 2005).

Again the mechanism of reduction in CNS Aβ was not clear. One hypothesis was that systemic antibodies were binding Aβ in the blood, drawing monomeric Aβ from the brain to the blood thus reducing the accumulation in the brain. This has commonly been referred to as the “sink hypothesis”. The alternative hypothesis concerns the direct action of the antibodies in the central nervous system. Antibodies binding directly to Aβ might target the protein for phagocytosis. Alternatively, antibody binding to Aβ could prevent Aβ aggregation or uptake by neurons.

The promising mouse data from the passive immunization prompted investigators to move this therapeutic approach to the clinic. Several anti-Aβ antibodies have been tested in clinical trials: bapineuzumab, solanezumab, gantenerumab, crenezumab, ponezumab among others in early clinical trials (Figure 1, Table 1).

**Table 1:** **Summary of the most advanced passive immunotherapies for Alzheimer’s disease**.

**Antibody**	**Source**	**Isotype**	**Epitope**	**Conformation**
**Bapineuzumab**	Humanized	IgG1	1–5	Fibrils/plaques
**Solanezumab**	Humanized	IgG1	13–28	Monomer
**Gantenerumab**	Human^1^	IgG1	1–11	Plaques
**Crenezumab**	Humanized	IgG4	12–23	Monomer, oligomer, fibrils
**Ponezumab**	Humanized	IgG2a	33–40	Monomer, plaques

*^1^phage derived*.

Bapineuzumab was the first passive immunotherapy in clinical trials for AD. The humanized antibody was developed against Aβ1-5 and was reported to bind to both amyloid fibrils as well as plaques. Clinical trials showed little cognitive improvement in patients with some patients receiving the high dose experiencing vasogenic cerebral edema (Salloway et al., 2014). These patients did recover, however clinical endpoints only showed modest reduction in CSF Tau and no reduction in CSF Aβ so trials were discontinued (Blennow et al., 2012). Further analysis of the data from the clinical trial did reveal some cognitive and functional benefits only in a subset of patients (Tayeb et al., 2013; Salloway et al., 2014). These patients were ApoE4 non-carriers. ApoE4 is an allele of the ApoE gene involved in cholesterol transport that is associated with an increased risk of AD.

Similar to bapineuzumab, solanezumab is a humanized antibody; however it is targeted to an internal epitope of Aβ (13–28). In addition, the antibody showed preferential binding to soluble Aβ but not fibrillar Aβ. Clinical trials showed increased plasma and CSF levels of Aβ following a dose dependent administration of solanezumab to patients in contrast to the trials with bapineuzumab. Early clinical trails showed little improvement in cognition in patients with moderate AD (Doody et al., 2014), however patients with mild AD showed a 33% reduction in a rate of decline so a phase III trial has begun to investigate the treatment of mild AD patients with solanezumab (NIH, 2014c). Early clinical trials have shown solanezumab has similar efficacy in patients with or without the ApoE4 allele (Samadi and Sultzer, 2011) in contrast to the bapineuzumab trial.

Gantenerumab is the only fully human antibody developed. It is targeted to Aβ1-11 and appears to bind preferentially to amyloid plaques and not to soluble amyloid. In animal models, treatment with gantenerumab reduces brain amyloid loads without increasing plasma Aβ levels as observed with solanezumab. The likely mode of action of this antibody appears to be binding to small plaques and inducing a phagocytic response by microglia (Bohrmann et al., 2012). Clinical trials in mild to moderate AD patients treated with gantenerumab reduced brain amyloid load by up to 30% as determined by PET scan, however two patients in the high dose group experienced vasogenic cerebral edema (Ostrowitzki et al., 2012).

Crenezumab is a humanized antibody targeted to Aβ with a modified human IgG isotype IgG4 to reduce the affinity for Fc receptor binding and reduce the risk of immune cell stimulation allowing higher dosing of the antibody than previous immunotherapies had allowed. Crenezumab appears to bind to many forms of Aβ including monomer, oligomer and fibrillar (Adolfsson et al., 2012). Phase I trials showed good safety data and now crenezumab is moving into Phase II testing.

Ponezumab is another humanized IgG antibody; however unlike other antibodies tested to date, ponezumab targets Aβ_40_ without targeting the full-length APP protein (Freeman et al., 2012a,b; La Porte et al., 2012). Early clinical data pointed to good safety profile with increased plasma Aβ_40_; however, little to no improvement in cognitive impairment was observed and trials were discontinued (Burstein et al., 2013; Landen et al., 2013). Currently, ponezumab is being examined for the treatment of cerebral amyloid angiopathy (CAA; NIH, 2014d).

Although many of these immunotherapeutic approaches have failed to affect significant improvements in the mild to moderate AD patients being treated, the exact cause of failure is not known. Two, not exclusive, hypotheses have been promoted to explain the lack of success: (1) poor uptake of monoclonal antibody to the CNS across the BBB; and (2) treatment of patients too late in the progression of AD. The first point will be addressed below. The topic of treating late stage patients with current mild to moderate cognitive impairment is important to take into account when reviewing the results of these trials. It has long been thought that during the later stages of AD, many of the neurodegenerative changes have occurred and reducing or eliminating Aβ accumulation at this stage may not be enough to overcome the deficit of neuronal functional loss. Treatment of patients earlier in diagnosis may be able to slow or stop the progression of the disease. The difficulty has always been in identifying patients early in the disease.

Three new trials of passive immunotherapy will attempt to investigate whether treatment at earlier stages of the disease can slow or stop the progression of AD. The Dominantly Inherited Alzheimer’s Network (DIAN) will treat a group of individuals identified to be carriers of the dominantly inherited familial Alzheimer’s disease (FAD) gene (Bateman et al., 2012). These patients are highly likely to suffer from early onset AD. Treatment will include solanezumab and gantenerumab in a phase II/III trial examining CSF Aβ and PET scan end points (NIH, 2014b).

The Alzheimer’s Prevention Initiative (API) follows large groups of families in Colombia that are known carriers of the FAD (Reiman et al., 2011). 300 people will be treated with crenezumab with the expectation that 2/3 will develop some form of AD in the future (Garber, 2012). Patients will be followed for changes in biomarkers for 5 years.

Finally, a study with the Treatment of Asymptomatic Alzheimer’s (A4) will examine older patients that are not known carriers of gene mutations for AD, but who show early signs of Aβ deposits determined by PET scans with no cognitive impairment. Patients will be treated with solanezumab to determine if early intervention, prior to the onset of cognitive decline, can prevent or delay AD symptoms (Carrillo et al., 2013).

## Future directions

The BBB controls the passage of substances from the blood into the central nervous system. Thus, a major challenge for the delivery of monoclonal antibodies following either active or passive immunization is the transport of these large proteins to the CNS.

Previous studies with monoclonal antibodies for the treatment of AD have shown approximately 0.1% of injected antibodies cross the BBB with the rest either metabolized in the liver or excreted through the kidneys (Banks et al., 2002). This low rate of transport may at least partially account for the low clinical success rate of several anti-Aβ therapies. In contrast, we, and others have shown that targeting receptors on the BBB for active transport or transytosis of protein to the CNS from the blood can increase the brain penetration to 2–3% of injected dose (personal observation, (Boado et al., 2013)). This increased BBB transport may be sufficient to move some of the current antibodies from clinically ineffective to effective.

The receptors expressed on the endothelial cells of the BBB function to capture proteins in the blood, endocytose the protein/ receptor complex and trancytose the complex to the neuronal side of the cells where the protein is released. Starzyk et al. were the first to show that targeting a receptor on the BBB could transport a “cargo” protein to the neuronal side of the BBB (Friden et al., 1991). An antibody developed against the transferrin receptor expressed on the BBB was able to transport methotrexate to the CNS. This same approach has been used to target the transport of proteins and peptides across the BBB efficiently (Shin et al., 1995; Boado et al., 2007).

**Figure 2:**
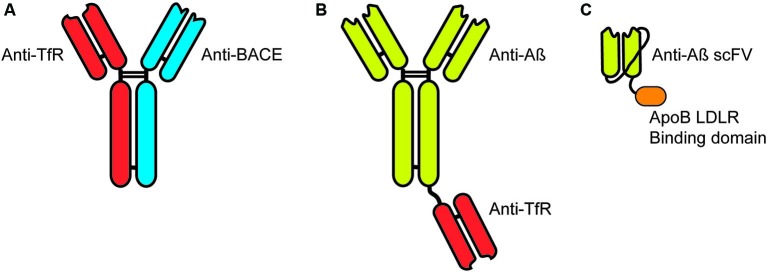
**Targeting immunotherapies for transport across the blood-brain barrier**. **(A)** Bi-specific IgG antibodies targeting the transferrin receptor (TfR) and β-secretase (BACE). **(B)** Bi-functional IgG antibodies target Aβ with a single Fab fragment targeting the TfR fused by the knobs into holes approach. **(C)** Single chain antibody (scFV) targeted to Aβ expressed with a linker between the heavy and light chain with a linker to the LDLR binding domain of Apolipoprotein B (38 amino acids).

More recently, this approach has been investigated in the context of delivering monoclonal antibodies for passive vaccination immunotherapy for AD. A bi-specific IgG developed to target the transferrin receptor with one Fab and the β-secretase (BACE1) with the other Fab (Figure 2A) resulted in increased brain penetration while significantly reducing brain Aβ levels (Couch et al., 2013). The breakthrough in these results was in the affinity of the Fab to the transferrin receptor (Yu et al., 2011). Too high an affinity resulted in antibody that remained bound to the transferrin receptor on the endothelial cell following trancytosis. This failed to provide significant levels of antibody in the brain parenchyma and failed to generate significant penetration of the recombinant targeting antibody. In contrast, lower affinity Fab targeted to the transferrin receptor actually increased the concentration and penetration of targeted antibody without altering BBB levels of the transferrin receptor (Bien-Ly et al., 2014).

Similarly, a bi-functional antibody developed with a full IgG targeted to the Aβ protein fused at the Fc region to a Fab targeted to the transferrin receptor (Figure 2B; Niewoehner et al., 2014). This bi-functional antibody penetrated the brain parenchyma significantly greater than the anti-Aβ monoclonal antibody alone and showed a significantly greater reduction in accumulated Aβ, while given at equal molar doses.

We have also targeted receptors on the BBB for the transport of proteins to the CNS. We utilized the low-density lipoprotein receptor (LDLR) expressed on the endothelial cells for transport to the CNS. In contrast to the methods listed above, we have used the endogenous LDLR binding domain of native proteins for the binding/transytosis of targeted proteins. These receptor binding domain have been identified from Apolipoprotein B (38 amino acids) (Spencer and Verma, 2007) and Apolipoprotein E (19 amino acids) (Spencer and Verma, 2007; Böckenhoff et al., 2014) and can be readily fused to target proteins including antibodies. Recently, we fused the 38 amino acid LDL-R binding domain of ApoB to a single-chain antibody (scFV) (Figure 2C). Addition of the LDLR binding domain significantly increased brain penetration of the scFV but also presented an unique cellular uptake and clearance mechanism (Spencer et al., unpublished).

Although it is not clear how monoclonal antibodies are cleared from the CNS following binding to their targeted protein, one mechanism is thought to be through the Fc receptor expressed on microglial cells. This could lead to cell mediated cytotoxicity and release of inflammatory mediators (Reviewed in Okun et al., 2010). We found the addition of the LDLR binding domain of ApoB, in addition to improving BBB transport, facilitated neuronal and glial cell uptake through the LDLR receptor leading to endosomal sorting complex required for transport (ESCRT) mediated endocytosis and autophagosome degradation thus bypassing the Fc receptor signaling cascade that could lead to cytotoxicity (Spencer et al., unpublished). scFV antibodies targeted to Aβ could be used with this approach to target AD (Liu et al., 2004; Fukuchi et al., 2006; Kasturirangan et al., 2009; Kasturirangan and Sierks, 2010).

Other approaches to targeting the brain and active transport across the BBB have been identified and are actively being investigated. These may prove to be synergistic to the field of immunotherapy and need to be followed closely. Clearly the techniques mentioned above show that there are novel methods for improving the concentration and penetration of immunotherapeutic antibodies in the CNS. This would have the benefit of not only increasing delivery of the injected dose but also possibly reducing the amount of antibody needed for injection thus reducing drug exposure and improving the safety window.

Aβ immunotherapy for the treatment of AD continues to show promise. After the setback from the early trials involving active immunization of full length Aβ_42_, current active immunization with N-terminus Aβ and passive immunization with monoclonal antibodies targeted to Aβ appear to show much greater promise with larger safety margins. Still, clinical efficacy has been difficult to achieve with the current approaches and theories as to why that has occurred include (1) late stage disease treatment; and (2) poor penetration of antibody to the brain. Both of these theories are being addressed now; the first with the trials involving treatment of individuals prior to onset of symptoms, and the second with the development in the lab of new strategies to improve the transport and penetration of antibodies to the CNS. The current standard of care for AD provides a modest transient relief of symptoms, however targeting the source of toxicity on AD may allow for a treatment or even cure if begun early enough in the disease course.

## Conflict of interest statement

The authors declare that the research was conducted in the absence of any commercial or financial relationships that could be construed as a potential conflict of interest.
